# Prevalence of trachoma in school children in the Marajó Archipelago, Brazilian Amazon, and the impact of the introduction of educational and preventive measures on the disease over eight years

**DOI:** 10.1371/journal.pntd.0006282

**Published:** 2018-02-15

**Authors:** Joana Favacho, Antonio José Ledo Alves da Cunha, Samara Tatielle Monteiro Gomes, Felipe Bonfim Freitas, Maria Alice Freitas Queiroz, Antonio Carlos Rosário Vallinoto, Ricardo Ishak, Marluísa de Oliveira Guimarães Ishak

**Affiliations:** 1 Health Surveillance Department, Evandro Chagas Institute, Bélem, Pará, Brasil; 2 Faculty of Medicine and Institute of Public Health, Federal University of Rio de Janeiro, Rio de Janeiro, Brasil; 3 Biological Sciences Institute, Federal University of Pará, Belém, Pará, Brasil; RTI International, UNITED STATES

## Abstract

Trachoma is the leading infectious cause of blindness in the world and is associated with precarious living conditions in developing countries. The aim of the present study was to evaluate the prevalence of trachoma in three municipalities of the Marajó Archipelago, located in the state of Pará, Brazil. In 2008, 2,054 schoolchildren from the public primary school system of the urban area of the region and their communicants were clinically examined; in 2016, 1,502 schoolchildren were examined. The positive cases seen during the clinical evaluation were confirmed by direct immunofluorescence (DIF) laboratory tests. The presence of antibodies against the genus *Chlamydia* was evaluated by indirect immunofluorescence (IIF), and the serotypes were determined by microimmunofluorescence (MIF). In 2008, the prevalence of trachoma among schoolchildren was 3.4% (69 cases) and it was more frequent in children between six and nine years of age and in females; among the communicants, a prevalence of 16.5% was observed. In 2016, three cases of trachoma were diagnosed (prevalence of 0.2%), found only in the municipality of Soure. The results of the present study showed that in 2008, trachoma had a low prevalence (3.4%) among schoolchildren in the urban area of Marajó Archipelago; eight years after the first evaluation and the introduction of control and prevention measures (SAFE strategy), there was a drastic reduction in the number of cases (0.2%), demonstrating the need for constant monitoring and effective measures for the elimination of trachoma.

## Author summary

Trachoma is one of the main neglected infectious diseases and carry a considerable burden to human health as a consequence of the clinical severity of the disease which may evolve to blindness. The lack of hygiene, education and other indicators of low social and economic markers occurring in developing and underdeveloped countries favour the spread of *Chlamydia trachomatis*, the bacterium causing trachoma. Although there is an easy, cheap and available treatment, reinfections are common and transmission is a consequence of bad hygienic habits and the various serotypes of the bacterium. The Marajó territory, in the North of the Amazon region of Brazil, is a large area with an ill educated, poor population, with almost no access to health resources and with almost no chance of transportation to major urban centers. Trachoma was detected a long time ago in the island and now, for the first time, a clear effort was produced during an eight year period in order to improve health hygienic habits among children and their relatives. The number of new cases following an initial diagnosis, was significantly reduced by the application of the WHO SAFE (Surgery, Antibiotics, Facial hygiene and Education for better habits) strategy.

## Introduction

Trachoma, an eye disease caused by *Chlamydia trachomatis* (serotypes A, B, Ba, and C), continues to be the leading infectious cause of blindness in the world [1,2]. The clinical presentation of trachoma is characterized by follicular and papillary hyperplasia in the conjunctiva, forming grayish-yellow follicles; the development of scars on the eyelid conjunctiva; trichiasis (inverted lashes touching the eyeball); and corneal opacity (which includes formation of pannus, epithelial vascularization, and infiltration), responsible for the blindness stage of trachoma [3].

The disease occurs mainly in places with precarious and crowded living conditions, poor sanitation, and low educational and cultural levels, which favors its direct (eye to eye or via contaminated hands) or indirect (including clothing and insect vectors) transmission. It is considered a household infection with higher incidence in childhood, which facilitates reinfections [2,4,5].

Currently, trachoma is a public health problem in 42 countries and is responsible for the blindness or visual impairment of approximately 1.9 million people. Almost 182 million people live in areas endemic for trachoma and are at risk of developing blindness [1].

In Brazil, serological evidence of the infection indicates that serotype A of *C*. *trachomatis* was introduced in the Amazon region during the eighteenth century from a community of immigrants from North Africa who settled in the region [6]. A study evaluating the presence of trachoma among Brazilian schoolchildren revealed a prevalence of 5%, and the states of Ceará, Acre, and Pará presented the highest prevalence rates (8.7%, 7.9%, and 6.6%, respectively) [7]. The state of Pará, an extensive geographic area located in the Amazon region, contains municipalities with prevalence rates of trachoma varying from zero to 29.4% [8]. However, some municipalities were not evaluated in the last surveillance surveys conducted in the state, including the 15 municipalities that are part of the Marajó Archipelago [8], although this region is one of the poorest in the state, exhibiting one of the lowest human development indices (HDI) in the country [9].

The World Health Organization (WHO) leads an international alliance for the elimination of trachoma by the year 2020 (GET 2020), which proposed recommendations and guidelines for collective and specific treatments, through the SAFE strategy (Surgery for entropion/trichiasis, Antibiotics for infectious trachoma, Facial cleanliness to reduce transmission, and Environmental improvements), which includes surgery, antibiotic therapy, constant facial hygiene and environmental improvements, and health education for changes in habits [10]. Brazil is part of this alliance and adheres to the elimination targets, working in conjunction with the state agencies coordinating trachoma surveillance and control programs [11].

The objective of the present study was to evaluate the prevalence of trachoma in the Marajó Archipelago in 2008 and 2016 and the impact of the introduction of educational and preventive measures on the disease during an eight-year interval.

## Methods

### Population characterization

**Fig 1:**
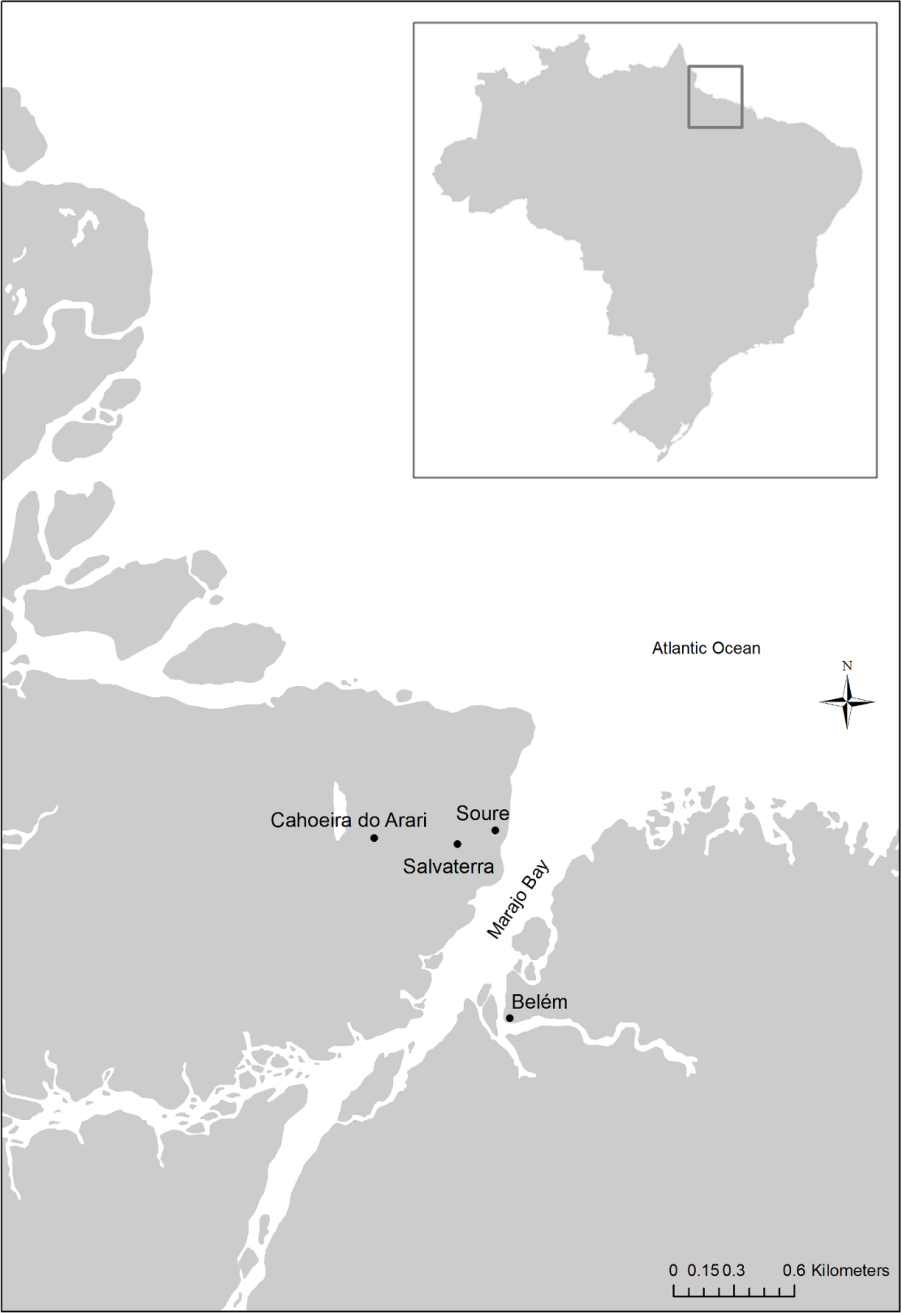
Geographic location of the municipalities of the Marajó Archipelago included in the study in relation to the city of Belém, capital of the state of Pará.

The study was conducted in three municipalities of the Marajó Archipelago, located in the state of Pará, in the Eastern Amazon estuary, considered the world's largest fluvial-maritime archipelago [12]. The areas involved in the present study were selected based in a previous study [13] which detected a large dissemination of trachoma among the children population. Seroepidemiological information showed a high prevalence of antibodies to *C*. *trachomatis* [14,15] in several areas of the Amazon region of Brazil which is commonly found among deprived population groups with a low HDI. The Marajo Archipelago is almost uniform in its poverty index and lack of health access and, indeed, the lowest HDI in Brazil is found in this geographical area. Fig 1 shows the geographic locations of the three municipalities included in the study, Soure, Salvaterra, and Cachoeira do Arari.

### Sample characterization and selection

A draw was performed to randomly select 16 schools visited from the municipalities of Soure (n = 7), Salvaterra (n = 6) and Cachoeira do Arari (n = 3). They were located in the urban areas because of the absence of regular travelling facilities within the Marajo. There were approximately 13,210 registered children in school during the period of examination, and the study included 2,054 children from 6 to 14 years, attending the first four school years. There were no revisits for missing children as they were present during the period of sample collection. The visits were preceeded by an extensive discussion with the community to ensure their presence during the investigation. A certified nurse trained by the Brazilian Ministry of Health program against trachoma conducted the examinations and the grading system for classification was that of Thylefors et al (1987) [16].

In 2008, the study evaluated 2,054 children of both sexes, attending public primary schools located in the urban area, of the municipalities of Soure (922 children), Salvaterra (572), and Cachoeira do Arari (560). The initial evaluation consisted of a clinical examination of both eyes using a 2.5x magnifying glass under sunlight and light from a flashlight, a common protocol used in field work, and children with severe disease were referred to a medical service in ophthalmology at the Universidade Federal do Para. The grading system for classification was that of Thylefors et al., 1987 [16]. The clinical cases were compared with photograph grading cards of the WHO Trachoma Program for the prevention of blindness and deafness and the cases were confirmed by a second examiner, following the instructions of the Brazilian Ministry of Health. The children who presented clinical diagnosis of trachoma were addressed for confirmation of the laboratory diagnosis.

The individuals responsible for the diseased children answered an epidemiological questionnaire. All communicants (individuals who had household contact with the children) who presented clinical signs of trachoma were also evaluated through clinical and laboratory examinations.

In March 2016, a new evaluation was conducted in the same schools of the three municipalities evaluated in 2008. A total of 1,502 children were evaluated, of whom 754 resided in Soure, 533 in Salvaterra, and 215 in Cachoeira do Arari. The epidemiological information was collected, and the clinical and laboratory tests were performed in the same way during the two investigative periods.

### Sample collection

Clinical cases of trachoma were defined as the presence of five or more follicles in the upper tarsal conjunctiva (trachomatous inflammation follicular–TF), marked inflammatory thickening of the conjunctiva with obstruction of more than half of the deep tarsal vessels (trachomatous inflammatory intense–TI), scars on the upper tarsal conjunctiva (trachomatous scarring–TS), inverted lashes touching the eyeball (trachomatous trichiasis–TT), and corneal opacity with reduced visual acuity (trachomatous corneal opacification–TCO) [17,18]. Children were classified as presenting a clean face when it was not possible to detect secretions either in the eyes or in the nose.

To confirm trachoma in children who presented clinical signs, blood samples and tarsal conjunctival scrapings were collected. A total of 5 mL of blood was collected by intravenous puncture into a tube with anticoagulant to obtain serum, which was used to detect antibodies to the genus *Chlamydia* and to the serotypes of *C*. *trachomatis*. The conjunctival scrapings were deposited on slides and were used for the detection of elementary body antigens of *C*. *trachomatis*. The samples were stored at -20°C and were transported to the Virology Laboratory, Biological Sciences Institute, Federal University of Pará and to the STD/Trachoma Laboratory, Bacteriology Section, Evandro Chagas Institute/Health Surveillance Department/Ministry of Health, where the laboratory tests were performed.

### Laboratory tests

#### Direct immunofluorescence (DIF) test

The DIF technique was performed using the conjunctival scrapings of the upper eyelid when everted. The smears were fixed on a slide with two drops of methanol and were dried for five minutes at room temperature. The slides were then incubated with fluorescein-conjugated monoclonal antibodies (Pathfinder Chlamydia trachomatis direct specimen kit, Bio-Rad, Hercules, California, USA) directed against the major outer membrane protein (MOMP), present in elementary bodies (EB) of *Chlamydia trachomatis* serotypes [19,20]. The technique consists of identifying the number of EBs and their relationship to the number of epithelial cells. In the present study, the presence of five or more EB in slides containing at least 100 epithelial cells was considered a positive case.

#### Indirect immunofluorescence (IIF) test

The presence of IgG antibodies to *Chlamydia* in the blood samples was detected by means of the IIF technique, performed according to a previously described protocol [15], which included the use of commercial slides impregnated with *C*. *trachomatis* serotype L_2_ antigens (BioMerieux, Paris, France), which react with all species of the genus *Chlamydia* [21].

#### Microimmunofluorescence (MIF) test

The MIF technique was used to identify the presence of antibodies to serotypes A, B, Ba, and C of *C*. *trachomatis* by preparing slides with antigens (EB) of the serotypes of interest (Washington Research Foundation, Seattle, Washington, USA). Samples were initially diluted 1:8 with phosphate-buffered saline (PBS) and were tested according to a previously standardized method [14]. Samples reacting against more than one serotype, were further diluted up to 512, to show the most probable reaction to the last serotype infection experienced by the host. The samples that showed clear, crisp, and greenish fluorescence against any of the serotypes were considered to be positive.

### Control and prevention measures

In the evaluation conducted in 2008, educational actions were developed to eliminate trachoma in the region, which included guideline instructions for the use of health staff and parents of the students for the adequate treatment of patients and the importance of constant facial cleaning of patients and communicants. Lectures were also held for the community dealing with preventive measures against trachoma, such as the adoption of hygienic practices in the elimination of infectious diseases, personal and environmental hygiene, avoiding the attraction of disease-transmitting animals, drinking boiled water, and adequate storage of food.

All identified cases of active trachoma were treated with 20 mg/kg (up to 1 g) of oral azithromycin in a single dose, as recommended by the Brazilian Ministry of Health [17].

### Statistical analysis

The information was collected and a data bank was prepared. Prevalence rates were calculated as usual using the number of persons with the characteristic involved over the total number of persons. Percentages were calculated using the rate between the number of persons with the characteristic and the total examined. When applicable, the binomial test was used for the comparison of two proportions.

### Ethical considerations

All study participants were informed about the objectives of the study, and the parents who agreed authorized the participation of the children by signing an informed consent form. The project was submitted and approved by the ethics committee on research with human beings of the Evandro Chagas Institute (protocols n. 0008/08 and n. 1.400.011).

## Results

In 2008, 1,030 (50.14%) of the 2,054 children investigated were males with an age range from six to 16 years. The prevalence of clinically diagnosed trachoma was 3.4% (69/2,054), all of them presenting the follicular form of the disease; most of the children had clean faces. The age of the children with trachoma (Table 1) ranged from 6 to 14 years, most were females, and the highest number of cases was seen in the municipality of Soure. The majority of the children lived in homes with four rooms, where they lived with six to nine persons, with a family income that varied between one and three times the minimum wage.

**Table 1:** Demographic and social characteristics of the children with clinical diagnosis of trachoma in the Marajó Archipelago, 2008.

Characteristics	N	%
**Age**		
6–9 years	39	56.53
10–14 years	30	43.47
**Sex**		
Male	23	33.33
Female	46	66.67
**Municipality**		
Cachoeira do Arari	19	27.54
Salvaterra	23	33.33
Soure	27	39.13
**Clean face**		
Yes	59	85.51
No	10	14.49
**Number of rooms in the home**		
1–2	11	15.94
3	14	20.29
4	35	50.73
5	9	13.04
**Number of residents**		
2–3	7	10.15
4–5	33	47.82
6–7	23	33.34
8–9	6	8.69
**Family income (minimum wage)**		
< 1	16	23.19
1–3	43	62.32
> 3	10	14.49

N: number of individuals.

Environmental variables which are commonly associated with the presence of trachoma were also present as part of the daily routine of the diseased children. There were plenty of domestic animals, garbage collection was not regular and all had contact with flies and mosquitoes. It was quite a homogeneous situation commonly involving also the non-diseased persons.

DIF tests confirmed the 69 clinical cases and all presented antibodies to the genus *Chlamydia*. The MIF test showed that most of the children in the three municipalities investigated were infected or have experienced previous infections by the four serotypes responsible for causing trachoma (Table 2).

**Table 2:** Frequency of antibodies to serotypes of *C*. *trachomatis* found in the investigated children of the municipalities in the Marajó Archipelago, 2008.

Serotype	Soure	Salvaterra	Cachoeira do Arari
	N = 27	N = 23	N = 19
	n (%)	n (%)	n (%)
A	27 (100)	19 (82.60)	19 (100)
B	27 (100)	20 (87)	19 (100)
Ba	27 (100)	22 (96)	18 (94.73)
C	27 (100)	20 (87	19 (100)

N: numbers of individuals.

Among the 243 communicants of the schoolchildren investigated in 2008, 40 (16.5%) had clinical and laboratory diagnosis of trachoma. The ages of the communicants ranged from six months to 94 years, and the majority were female, residing in the municipality of Salvaterra. The trachomatous FT form was the most evidenced in children (95%, 38/40), while the ST form was most evidenced in adults (5%, 2/40). The socio-demographic characteristics related to the communicants are listed in Table 3.

**Table 3:** Variables related to the communicants of children diagnosed with trachoma in the Marajó Archipelago, 2008.

Characteristics	N	%
Age (years)		
1–9	7	17.5
> 10	33	82.5
**Sex**		
Male	16	40
Female	24	60
**Municipality**		
Cachoeira do Arari	5	12.5
Salvaterra	22	55
Soure	13	32.5

N: numbers of individuals.

In 2016, 1,502 school children were examined in the three municipalities (Cachoeira do Arari, Salvaterra and Soure), and three cases of trachoma (0.2%) were found in Soure, which presented the FT form; none of the 13 communicants presented clinical suspicion of disease. The ages of the children with trachoma varied between seven and eight years, and all of them were female and resided in Soure. Five to six individuals lived in the households of these children, and the family income ranged from less than one to three times the minimum wage. The visits occurred in the months of June (in 2008) and March (2016) when rain precipitation, humidity and river conditions were similar.

## Discussion

WHO estimates that the prevalence of trachoma, including trichiasis and blindness, has decreased from 317 million in 2010 to 200 million in 2016 as a result of the implementation of the SAFE strategy [22].

The present study evaluated the influence of control and prevention measures on the prevalence rates of the disease in 2008 and 2016 in schoolchildren from three municipalities of the Marajó Archipelago, a geographical area with rural economic activity and a low development index [9].

The prevalence of trachoma in the initial visit in 2008 (3.4%) was similar to that reported among schoolchildren in other regions of Brazil (5.0%) [7], but it was below the prevalence found in the municipality of Cachoeira do Arari in a collection performed in 2007 (24.1%) in children aged from 0–15 years [13]. In other studies performed in the Amazon region, high prevalence rates among Indians of the upper Rio Negro region (28.5%) [23] and among Xingu Indians (28%) were identified [24], differing from the rates found in this study, which were similar to the prevalence rates recorded in other states, such as Maranhão (4.1%), Tocantins (5.6%), Bahia (3.5%), Goiás (5.2%), and São Paulo (4.1%) [7]. The difference in the prevalence rates of trachoma among these studies is possibly related to the location where the studies were conducted, since those in rural communities reported higher prevalence rates, whereas those studies performed in the urban parts of the cities or nearby found lower prevalence rates. Trachoma is one of the leading causes of blindness in many developing countries, especially in poor rural areas [25].

All of the diseased children presented the follicular form of trachoma, which is most commonly found among children and adolescents [7,22,26]. This clinical form is characterized by a mild and self-limited but highly contagious disease that may be responsible for the maintenance of infection in a community and the cause of reinfections, leading to the development of more severe forms of trachoma [17]. Children are the main reservoir of infection [27], and as they grow older there is a significant risk factor for the development of trichiasis, corneal opacity, and visual loss [28]. Thus, the correct diagnosis of the disease and treatment of children are important for the elimination of the most severe forms and the possible eradication of the disease.

The main age group affected with trachoma includes children aged one to five years [4]. Although the present study did not evaluate children in this age group, a high frequency of infection was observed among children aged six to nine years, which corroborates the suggestion of decreased frequency of trachoma with increasing age [29,30,31]. The frequency of trachoma was twice as high in females, differing from the frequencies found in other regions of Brazil, where the prevalence was higher among males or did not differ in its prevalence according to sex [7,29,31]. It is possible that the greater frequency of trachoma cases among females could be related to the more affective behavior among them, as direct person-person contact is an important form of infection transmission [2].

The majority of schoolchildren with trachoma presented clean faces, but they reported living with large numbers of people and did not enjoy a standard of living that kept them away from crowding, hygienic standards and other conditions which would lead them to a healthier life. These conditions favour the occurrence of *C*. *trachomatis* infection in the region. Trachoma is associated with precarious living conditions, poverty, lack of hygiene, and poor housing and sanitation conditions [4,29].

The transmission of trachoma may be maintained via the sharing of domestic and peridomestic environment among infected individuals and of these with animals such as buffaloes, horses, dogs and birds, among other animals [2,32] such as those of the Marajó fauna, which can lead to an unhealthy environment, attracting winged insects often implicated as vectors of bacterial conjunctivitis, including eye gnats and flies (*Liohippelates* and *Hippelates*) [33]. The mechanical transmission of *C*. *trachomatis* occurs when these mosquitoes land on the face of the diseased children with trachoma and transfer the bacteria to others, closing the transmission cycle [5]. It is important to emphasize this form of transmission because of the large number of vectors and the finding that all infected children had constant contact with flies and other mosquitoes, as was previously shown regarding the involvement of synanthropic flies in the transmission of *C*. *trachomatis* and trachoma dissemination [13].

Most of the children investigated were positive for the four *C*. *trachomatis* serotypes associated with trachoma. All of these serotypes are usually present in developing countries where trachoma is endemic [34]. They are frequently associated with the precarious life conditions of the population, demonstrating the endemic character of the disease in Marajó Island, requiring a continuous monitoring program for trachoma prevention and treatment.

Secondary infections of the communicants showed a high prevalence of the disease with different presentations in children and adults, confirming the findings of the different forms of trachoma according to age. The clinical form, FT, decreases inversely to advancing age, in contrast to the prevalence of ST [3,29]. The presence of adults with scars among the communicants indicates that the individual was exposed to trachoma at a very young age and that the children with the FT form are possibly responsible for the maintenance of transmission [35].

The return to the same communities in 2016, eight years after the first investigation, showed a significant reduction in the prevalence of trachoma (0.2%). Three positive cases were diagnosed in one municipality (Soure), in one single school; the three children belonged to three different families in the municipality. The presence of these cases may be related to visits by the families to rural areas, which are usually difficult to reach and that have shown a high prevalence of trachoma [13].

The decrease in the prevalence of trachoma is associated with disease control and prevention measures enacted by the team involved in the study together with the communities. According to the recommendations of the WHO and the Trachoma Program of the Brazilian Ministry of Health, the SAFE strategy (Surgery, Antibiotics, Face, Environment) for the elimination of trachoma was tentatively applied [10]. Cases which were more severe were referred to a University hospital for surgery. Outpatients and their communicants were treated with antibiotics with the suggested regimens of the Trachoma Program. Health and educational authorities, nurses and community leaders of the municipalities were joined in a net with school teachers, parents and children who received educational talks with the distribution of folders and banners regarding the disease, and ways to prevent it, including the maintenance of clean faces to avoid bacterial transmission. Finally, environmental improvements were strongly stressed to the municipality net regarding individual hygiene, the appropriate use of latrines, the elimination of vectors, the water supply and public garbage disposal. The intervention had to be done by the municipality and the power of convincing the community certainly helped to decrease the number of trachoma cases eight years later. Although it was not possible to intervene directly with the authorities regarding public services, there was a change in their behavior regarding the prophylaxis of trachoma. The strategy may not have been applied in full, but it was certainly an enormous help to change the epidemiological distribution of trachoma in the area of the study.

It is also important to articulate measures ranging from combating poverty-related diseases to adopting public policies aimed at improving basic sanitation and environmental conditions [11,17]. In conclusion, the present study showed that trachoma presented a low prevalence (3.4%) among schoolchildren in the urban area of the Marajó Archipelago in 2008, and eight years later following the introduction of control and prevention measures, there was a drastic reduction in the number of cases (0.2%), emphasizing the need for constant monitoring and the implementation of effective measures for the elimination of trachoma.

## References

[1] World Health Organization. Trachoma. Fact sheet, update in april 2017. Available from: http://www.who.int/mediacentre/factsheets/fs382/en/

[2] Gambhir M, Basáñez MG, Turner F, Kumaresan J, Grassly NC. Trachoma: transmission, infection, and control. Lancet Infect Dis. 2007;7: 420–427. doi: 10.1016/S1473-3099(07)70137-8 17521595 10.1016/S1473-3099(07)70137-8

[3] Mohammadpour M, Abrishami M, Masoumi A, Hashemi H. Trachoma: Past, present and future. J Curr Ophthalmol. 2016;28: 165–169. doi: 10.1016/j.joco.2016.08.011 27830198 10.1016/j.joco.2016.08.011 PMC5093790

[4] Mariotti SP, Pascolini D, Rose-Nussbaumer J. Trachoma: global magnitude of a preventable cause of blindness. Br J Ophthalmol 2009;93: 563–568. doi: 10.1136/bjo.2008.148494 19098034 10.1136/bjo.2008.148494

[5] Emerson PM, Lindsay SW, Walraven GEL, Faal HB, Bogh C, Lowe K, et al Effect of fly control on trachoma and diarrhoea. Lancet 1999;353: 1401–1403. doi: 10.1016/S0140-6736(98)09158-2 10227221 10.1016/S0140-6736(98)09158-2

[6] Ishak M de O, Costa MM, Almeida NC, Santiago AM, Brito WB, Vallinoto AC, Azevedo VN, Ishak R. *Chlamydia trachomatis* serotype A infections in the Amazon region of Brazil: prevalence, entry and dissemination. Rev Soc Bras Med Trop. 2015;48: 170–174. doi: 10.1590/0037-8682-0038-2015 25992931 10.1590/0037-8682-0038-2015

[7] Lopes MFC, Luna EJA, Medina NH, Cardoso MRA, Freitas HSA, Koizomy IK, Bernardes NAFA, Guimarães JA. Prevalência de tracoma entre escolares brasileiros. Rev Saúde Pública 2013;47: 451–459. 24346557 10.1590/s0034-8910.2013047003428

[8] Brasil. Ministério da Saúde. Sistema nacional de vigilância em saúde: relatório de situação: Pará/Ministério da Saúde, Secretaria de Vigilância em Saúde 5. ed. Brasília: Ministério da Saúde, 2011 Available from: http://bvsms.saude.gov.br/bvs/publicacoes/pa1.pdf

[9] Atlas do Desenvolvimento Humano no Brasil. IDHM 2013. Available from: http://www.atlasbrasil.org.br/2013/pt/consulta/

[10] World Health Organization. Report of the 18th meeting of the WHO alliance for the global elimination of trachoma by 2020. Available from: http://apps.who.int/iris/bitstream/10665/163362/1/9789241508681_eng.pdf?ua=1

[11] Alves FAP, Souza WV, Luna CF, Gouveia GC. Análise das intervenções e dos fatores socioambientais associados à ocorrência de tracoma em Pernambuco a partir de dois inquéritos em escolares realizados em 2006 e 2012. Cad. Saúde Colet. 2016; 24: 435–442.

[12] Portal Brasil. Turismo. Marajó: jornada pelo maior arquipélago flúvio-marítimo do mundo. Available from http://www.brasil.gov.br/turismo/2014/11/marajo-jornada-pelo-maior-arquipelago-fluvio-maritimo-do-mundo.

[13] Reilly LA; Favacho J; Garcez LM; Courtenay O. Preliminary evidence that synanthropic flies contribute to the transmission of trachoma-causing *Chlamydia trachomatis* in Latin America. Cad Saúde Pública. 2007;7: 1682–1688. 10.1590/s0102-311x2007000700020 17572818

[14] Ishak MOG, Ishak R. O impacto da infecção por *Chlamydia* em populações indígenas da Amazônia brasileira. Cad Saúde Pública 2001;17: 385–396. 11283769 10.1590/s0102-311x2001000200013

[15] Ishak MOG, Ishak R, Cruz AC, Santos DE, Salgado U. Chlamydial infection in the Amazon region of Brazil. Trans R Soc Trop Med Hyg. 1993;87: 60–62. 8465397 10.1016/0035-9203(93)90421-l

[16] Thylefors B, Dawson CR, Jones BR, West SK, Taylor HR. A simple system for the assessment of trachoma and its complications. Bull World Health Organ. 1987;65: 477–483. 3500800 PMC2491032

[17] Brasil. Ministério da Saúde. Manual de vigilância do tracoma e sua eliminação como causa de cegueira / Ministério da Saúde, Secretaria de Vigilância em Saúde, Departamento de Vigilância das Doenças Transmissíveis– 2. ed.–Brasília: Ministério da Saúde, 2014 Available from: http://bvsms.saude.gov.br/bvs/publicacoes/manual_vigilancia_tracoma_eliminacao_cegueira.pdf

[18] Solomon AW, Zondervan M, Kuper H, Buchan JC, Mabey DCW, Foster A. Trachoma control: A guide for programme managers Geneva, Switzerland: World Health Organization, 2006.

[19] Stephens RS, Kuo CC, Tam MR. Sensitivity of immunofluorescence with monoclonal antibodies for detection of *Chlamydia trachomatis* inclusions in cell culture. J Clin Microbiol. 1982;16: 4–7. 6179965 10.1128/jcm.16.1.4-7.1982 PMC272285

[20] Medina NH, Gentil RM, Caraça M, Suzuki CK, Melles HHB. Analysis of direct immunofluorescence tests for trachoma diagnosis. Rev de Saúde Pública. 1996;30: 135–140. 10.1590/s0034-89101996000200004 9077011

[21] Treharne JD, Forsey T, Thomas BJ. Chlamydial serology. Br Med Bull 1983;39: 194–200. 6347331 10.1093/oxfordjournals.bmb.a071815

[22] World Health Organization. Global Health Observatory (GHO) data. Trachoma, Situation and trends. Available from: http://www.who.int/gho/neglected_diseases/trachoma/en/

[23] Reis ACPP, Chaves C, Cohen J.M, Belfort F, Oliveira NP, Belfort Júnior R. Detecção de tracoma e doenças corneanas em índios da região do Alto Rio Negro. Arq. Bras. Oftalmol 2002;65: 79–81.

[24] Garrido C, Guidugli T, Campos M. Estudo clínico-laboratorial do tracoma em população indígena da Amazônia brasileira. Arq. bras. oftalmol. 1999;62: 132–138.

[25] Hu VH, Harding-Esch EM, Burton MJ, Bailey RL, Kadimpeul J, et al Epidemiology and control of trachoma: systematic review. Trop Med Int Health. 2010;15: 673–691. doi: 10.1111/j.1365-3156.2010.02521.x 20374566 10.1111/j.1365-3156.2010.02521.x PMC3770928

[26] Koizumi IK, Medina NH, D'Amaral RK, Morimoto WT, Caligaris LS, Chinen N, Andrade YM, Cardoso MR. Prevalence of trachoma in preschool and schoolchildren in the city of São Paulo. Rev Saúde Pública. 2005;39: 937–942. 16341404 10.1590/s0034-89102005000600011

[27] Burton MJ, Mabey DC. The global burden of trachoma: a review. PLoS Negl Trop Dis. 2009;3: e460 doi: 10.1371/journal.pntd.0000460 19859534 10.1371/journal.pntd.0000460 PMC2761540

[28] Bowman RJ, Jatta B, Cham B, Bailey RL, Faal H, Myatt M, Foster A, Johnson GJ. Natural history of trachomatous scarring in The Gambia: results of a 12-year longitudinal follow-up. Ophthalmology. 2001;108(12): 2219–2224. 11733262 10.1016/s0161-6420(01)00645-5

[29] Lucena AR, Cruz AAV, Cavalcanti R. Estudo epidemiológico do tracoma em comunidade da Chapada do Araripe—Pernambuco–Brasil. Arq Bras Oftalmol. 2004;67: 197–200.

[30] Grassly NC, Ward ME, Ferris S, Mabey DC, Bailey RL. The natural history of trachoma infection and disease in a Gambian cohort with frequent follow-up. PLoS Negl Trop Dis. 2008;2: e341 doi: 10.1371/journal.pntd.0000341 19048024 10.1371/journal.pntd.0000341 PMC2584235

[31] Luna EJA, Lopes MFC, Medina NH, Favacho J, Cardoso MRA, Prevalence of trachoma in schoolchildren in Brazil. Ophthalmic Epidemiol. 2016;23: 360–365. doi: 10.1080/09286586.2016.1244274 27824506 10.1080/09286586.2016.1244274

[32] Vashist P, Gupta N, Rathore AS, Shah A, Singh S. Rapid assessment of trachoma in underserved population of Car-Nicobar Island, India. PLoS One. 2013;8: e65918 doi: 10.1371/journal.pone.0065918 23799063 10.1371/journal.pone.0065918 PMC3683059

[33] Tondella MLC, Paganelli CH, Bortolotto IM, Takano OA, Irino K, Brandileone MCC, et al Isolamento de *Haemophiliis aegyptius* associado à febre purpúrica brasileira, de cloropídeos (Diptera) dos gêneros *Hippelates e Liohippelates*. Rev Inst Med Trop São Paulo. 1994;36: 105–109. 7997783

[34] Hu VH, Holland MJ, Burton MJ.Trachoma: protective and pathogenic ocular immune responses to *Chlamydia trachomatis*. PLoS Negl Trop Dis. 2013;7: e2020 doi: 10.1371/journal.pntd.0002020 23457650 10.1371/journal.pntd.0002020 PMC3573101

[35] Solomon AW, Holland MJ, Burton MJ, West SK, Alexander ND, Aguirre A, Massae PA, Mkocha H, Muñoz B, Johnson GJ, Peeling RW, Bailey RL, Foster A, Mabey DC. Strategies for control of trachoma: observational study with quantitative PCR. Lancet. 2003;362:198–204. doi: 10.1016/S0140-6736(03)13909-8 12885481 10.1016/S0140-6736(03)13909-8

